# Biliary-intestinal anastomosis leads to alterations in intestinal flora and its flora metabolites and increases the risk of long-term postoperative complications: a case-control study

**DOI:** 10.3389/fmicb.2025.1531955

**Published:** 2025-02-26

**Authors:** Baicheng Li, Zhao Chen, Guangzhi Wang, Yuzhuo Chen, Xingdong Hou, Bowei Lu, Shili Ning

**Affiliations:** Department of General Surgery, The Second Hospital of Dalian Medical University, Dalian, China

**Keywords:** intestinal flora, flora metabolites, biliary-intestinal anastomosis, long-term postoperative complications, Escherichia-Shigella

## Abstract

**Objective:**

Pancreaticoduodenectomy (PD) is a major surgical intervention that encompasses the resection of multiple organs and the reconstruction of the digestive tract, with reconstructive procedures including pancreatico-enteric, bilioenteric, and gastroenteric anastomoses. Prior research has documented a high incidence of long-term complications following PD, which significantly impact patient prognosis and survival, however, the underlying mechanisms remain elusive. Evidence from previous studies suggests that biliary-intestinal anastomosis modifies biliary tract anatomy, altering bile flow into the gut and potentially affecting the gut microbiota and its metabolites. Given the close association between biliary tract infections and alterations in gut microbiota, we hypothesize that changes in intestinal flora and its metabolites post-PD may be a critical factor in the development of long-term complications. The objective of this study is to investigate whether biliary-intestinal anastomosis during PD induces changes in the intestinal microbiota and its metabolites, which in turn may increase the risk of long-term postoperative complications.

**Methods:**

This study included 17 patients who underwent the procedure (group T) and 20 sex- and age-matched controls who did not (group N), patients in group T were stratified into those with (complication group) and without (non-complication group) long-term postoperative complications. Faecal samples were collected from all subjects and DNA was extracted from the samples using 16S rRNA gene sequencing to analyse the composition of the faecal flora and detect flora metabolites.

**Results:**

1. Alpha diversity analysis of the two sample groups indicated a trend towards lower microbial abundance in Group T relative to Group N, however, no significant differences were observed in the Shannon and Simpson diversity indices. 2. At the genus level, Group T patients exhibited markedly higher levels of Escherichia-Shigella, Veillonella, and Enterobacter, while showing significantly lower abundance of Blautia and Bifidobacterium compared to Group N subjects. Analysis of Spearman’s correlation and degree of correlation between genera showed a significant negative correlation between Escherichia shigella and Blautia. Veillonella showed a significant positive correlation with both Escherichia shigella and Enterobacter. In addition, Blautia and Bifidobacterium showed a significant positive correlation with each other. 3. Subsequent comparative analysis of the bacterial flora between the complication and non-complication groups revealed a significantly elevated abundance of Escherichia-Shigella in the complication group as compared to the non-complication group. 4. Faecal metabolomic analysis revealed that L-palmitoylcarnitine, arachidic acid and PG 13:0_15:0 were significantly increased in the T group compared to the N group, whereas 3-isopropylmalic acid was significantly decreased in the T group. 5. KEGG pathway analysis identified nine crucial metabolic pathways associated with these microbial shifts: alterations in starch and sucrose metabolism, steroid hormone biosynthesis, caffeine metabolism, the citric acid cycle, riboflavin metabolism, sulfur metabolism, and the biosynthesis of valine, leucine, and isoleucine, as well as pyruvate metabolism and ABC transporter protein pathways.

**Conclusion:**

1. The biliary-intestinal anastomosis, which is performed as part of a pancreaticoduodenectomy, induces significant shifts in the intestinal flora. 2. Increased abundance of Escherichia-Shigella may promote long-term complications after biliary-intestinal anastomosis. 3. Biliary-intestinal anastomosis leads to alterations in the metabolites of the patient’s intestinal flora. 4. Intestinal flora and their metabolites in patients after biliary-intestinal anastomosis may contribute to the development of long-term complications through nine metabolic pathways.

## 1 Introduction

Pancreaticoduodenectomy (PD) is the principal surgical intervention for malignancies of the pancreatic head, lower bile duct, duodenum, and other perampullary cancers. This procedure entails the resection of multiple organs and the subsequent reconstruction of the digestive tract, involving reconstructive techniques such as pancreatico-enteric, biliary-enteric, and gastroenteric anastomoses (Li et al., 2024). Biliary-enteric anastomosis, a biliary-enteric shunt commonly performed by anastomosing the common bile duct to the jejunum post-cholecystectomy, is the predominant method for biliary tract reconstruction, May provide patients with a long-term stable flow of bile into the intestine. However, this anastomosis alters the bile ducts anatomy and modifies the flow of bile into the intestine compared to a bile duct-bile duct end-to-end anastomosis. Consequently, it has been hypothesized that biliary-enteric anastomosis may exert effects on the intestinal microbiota and their metabolites (Mittler et al., 2021; Toriigahara et al., 2023). These alterations may precipitate disruptions in the gut’s micro-ecological balance, potentially impacting human health.

The gut microbiome’s stability is intricately linked to human health, with gut dysbiosis strongly associated with a spectrum of diseases, including colorectal cancer (Yin et al., 2022), diabetes mellitus (Yang Y. et al., 2023), and hematological cancers (Hussein et al., 2024). Dysbiosis primarily manifests as functional alterations in the microbiome’s metabolome, proteome, and transcriptome (Ahearn-Ford et al., 2022). Gut microbiota dysbiosis has also been linked to a spectrum of gastrointestinal symptoms, highlighting the impact of microbial imbalance on gut health (Ringel and Carroll, 2009). Furthermore, certain surgical procedures can disrupt gut microbial homeostasis, leading to intestinal dysbiosis, which may be a crucial factor in the development of postoperative complications (Li Y. D. et al., 2021). Cholecystectomy serves as a pertinent example, numerous studies have confirmed that some patients may experience complications such as diarrhea and vomiting following the procedure. This is attributed to the disruption of the normal physiological rhythm of bile excretion into the intestine, affecting enterohepatic circulation and causing an imbalance in intestinal flora. Probiotic interventions have been shown to play a significant role in mitigating postoperative syndromes in response to the intestinal dysbiosis that can occur with such procedures (Di Ciaula et al., 2018; Yoon et al., 2019; Xu et al., 2023b; Chen et al., 2020; Xu et al., 2023a). Patients who undergo biliary-intestinal anastomosis often face long-term complications, such as biliary retrograde infection, pancreatitis (Yamguchi et al., 2023), and gastric retention (Li et al., 2023). Consequently, the normal physiological rhythm of bile excretion into the intestine in these patients is also likely to be disrupted, impacting enterohepatic circulation and potentially leading to intestinal dysbiosis. This dysbiosis may be a key factor in the development of long-term complications following biliary-intestinal anastomosis in some patients.

In this study, we aimed to evaluate the alterations in gut microbiota following biliary-intestinal anastomosis in patients undergoing pancreaticoduodenectomy. Fecal samples were collected, and 16S rRNA gene sequencing was employed to extract and analyze the DNA, assessing the faecal microbiota composition and metabolite profiles. We conducted comparative analyses to identify differences in microbiota and metabolites between patients who underwent biliary-intestinal anastomosis and a control group without surgery, revealing that anastomosis can induce intestinal microecological imbalances. Further analysis comparing the composition of the intestinal flora of patients who developed distant complications after biliary-intestinal anastomosis and those who did not, confirmed that dysbiosis of the intestinal flora may have an impact on the occurrence of postoperative distant complications. The objective of this study is to investigate whether biliary-intestinal anastomosis during PD induces changes in the intestinal microbiota and its metabolites, which in turn may increase the risk of long-term postoperative complications.

## 2 Materials and methods

### 2.1 Volunteer recruitment

A total of 83 volunteers were enrolled in this study, out of which 37 individuals fulfilled the inclusion criteria. The study cohort consisted of 17 patients who had undergone pancreaticoduodenectomy (Group T) and 20 sex- and age-matched controls who had not undergone the procedure (Group N). All participants in Group T had their surgery performed at the Department of General Surgery, Second Hospital, Dalian Medical University. Fecal samples from all subjects were collected over a period from January to April 2024.

### 2.2 Inclusion and exclusion criteria

The inclusion criteria for patients in Group T were as follows: (1) Ages ranging from 40 to 80 years, (2) No history of other gastrointestinal tumors, (3) Previous pancreaticoduodenectomy and no history of any other surgery of the gastrointestinal tract. (4) No use of antibiotics, probiotics, or similar interventions within the last month, (5) No presence of irritable bowel syndrome, inflammatory bowel disease, or related conditions, (6) Absence of chronic diseases such as severe heart failure, uncontrolled hypertension, or diabetes mellitus, (7) No administration of chemotherapy drugs within the past 6 months, (8) No evidence of tumor recurrence or metastasis.

The inclusion criteria for the patients in the N group were as follows: (1) Age range of 40 to 80 years, (2) No history of gastrointestinal tumors, (3) No prior pancreaticoduodenectomy or other gastrointestinal surgeries, (4) No consumption of antibiotics, probiotics, or similar interventions within the last month, (5) No diagnosis of irritable bowel syndrome, inflammatory bowel disease, or related conditions, (6) Absence of chronic diseases including severe heart failure, uncontrolled hypertension, or diabetes mellitus.

### 2.3 Specimen collection

To ensure the integrity and stability of the specimens, the following preservation measures were implemented: Fresh fecal samples were stored at −80°C upon collection.

### 2.4 DNA extraction

During the subsequent four weeks, the fecal samples underwent a meticulous DNA extraction procedure using the Omega E.Z.N.A Stool DNA Kit (Omega Bio-Tek, Inc., United States). This process was performed in strict adherence to the manufacturer’s detailed protocol.

### 2.5 16SrRNA gene sequencing of fecal samples

The V3 and V4 variable regions of the 16S rRNA gene were targeted for amplification using universal primers 341F (5’-CCT AYG GGR BGC ASC AG-3′) and 806R (5′-GGA CTA CNN GGG TAT CTA AT-3′). Each primer was equipped with a distinct 8-nucleotide barcode index to enable multiplexed sampling. PCR was conducted with New England Biolabs’ Phusion^®^ High-Fidelity PCR Master Mix with GC Buffer, leveraging high-fidelity enzymes to ensure both amplification efficiency and accuracy. The PCR mixture consisted of 2 × Taq PCR mix (25 μL), Primer F (10 μM, 1 μL), Primer R (10 μM, 1 μL), gDNA (2.5 μL), and H2O (8.0 μL). The PCR protocol involved an initial denaturation at 95°C for 5 min, followed by 34 cycles of 94°C for 1 min, 57°C for 45 s, and 72°C for 1 min, with a final extension at 72°C for 10 min and a hold at 16°C for 5 min. Following PCR cleanup and indexing, sequencing was carried out on the Illumina MiSeq platform in strict accordance with the manufacturer’s protocol.

### 2.6 16S rRNA gene compositional analysis

For data analysis, we employed the QIIME2 software package (version 2017.12), utilizing the DADA2 pipeline to meticulously filter out low-quality and potentially chimeric sequences. This approach facilitated the generation of unique and highly distinguishable amplicon sequence variants (ASVs). The sequences, precisely processed by DADA2, were grouped into operational taxonomic units (OTUs) with enhanced recognition accuracy, which we term sequence variants as they represent 100% of the OTUs. The capabilities of QIIME2 were further highlighted by its generation of comprehensive feature lists and representative sequence files. We observed significant variation in sequencing depth among samples. To refine the taxonomic classification, we mapped the sequences with 99% sequence identity to an optimized version of the GreenGenes database (version 13.8), which is tailored to the V3-V4 region, providing us with precise and detailed taxonomic insights.

### 2.7 Non-targeted metabolomics

#### 2.7.1 Metabolite extraction

##### 2.7.1.1 Tissue sample preparation

① Homogenize 100 mg of tissue sample using liquid nitrogen and transfer the powder to an EP tube. Add 500 μL of an 80% methanol solution in water to the tube. ② Vortex the mixture, then place it on an ice bath for 5 min followed by centrifugation at 15,000 g for 20 min at 4°C. ③ Transfer a portion of the supernatant to a new tube and dilute it with mass spectrometry-grade water to achieve a final methanol concentration of 53%. ④ Recentrifuge the diluted supernatant at 15,000 g for 20 min at 4°C, collect the supernatant, and proceed to LC–MS analysis for metabolite profiling (Want et al., 2013).

##### 2.7.1.2 For liquid samples

① Pipet 100 μL of the sample into an EP tube and add 400 μL of an 80% methanol solution. Subsequently, follow the extraction and processing steps ② through ④ as outlined for tissue samples (Want et al., 2006; Barri and Dragsted, 2013).

##### 2.7.1.3 Cellular and bacterial sample preparation

① Transfer cell or bacterial samples into EP tubes and add 300 μL of an 80% methanol solution in water. ② Freeze the samples in liquid nitrogen for 5 min, then thaw on ice and vortex for 30 s, followed by sonication for 6 min. ③ Centrifuge the samples at 5000 rpm at 4°C for 1 min, transfer the supernatant to a new centrifuge tube, and lyophilize to obtain a dry powder. ④ Reconstitute the dried samples in a 10% methanol solution and analyze using LC–MS (Sellick et al., 2011; Yuan et al., 2012).

##### 2.7.1.4 Culture medium supernatant and cellular bacterial culture fluid sample preparation

① Lyophilize 1 mL of the sample using a freeze-dryer, then add 100 μL of an 80% methanol solution. ② Vortex the mixture, place it on an ice bath for 5 min, and centrifuge at 15,000 g for 15 min at 4°C. ③ Transfer a portion of the supernatant to a new tube and dilute it with mass spectrometry-grade water to achieve a 53% methanol solution. ④ Collect the supernatant by centrifugation at 15,000 g for 15 min at 4°C and proceed to LC–MS analysis.

##### 2.7.1.5 QC Sample preparation

Pool an equal volume from each experimental sample to create a composite QC sample.

##### 2.7.1.6 Blank sample preparation

Replace the experimental sample with a 53% aqueous methanol solution. Subject the blank sample to the same pre-treatment process as the experimental samples.

#### 2.7.2 Instrument parameters

Chromatographic Conditions: Column: Hypersil Gold C18 column. Column Temperature: 40°C. Flow Rate: 0.2 mL/min. Positive Ion Mode: Mobile Phase A: 0.1% Formic Acid in Water. Mobile Phase B: Methanol. Negative Ion Mode: Mobile Phase A: 5 mM Ammonium Acetate, pH adjusted to 9.0. Mobile Phase B: Methanol (Table 1).

**Table 1:** Gradient elution procedure.

Time	A%	B%
0	98	2
1.5	98	2
3	15	85
10	0	100
10.1	98	2
11	98	2
12	98	2

#### 2.7.3 Mass spectrometry conditions

Scan Range: m/z 100–1,500. ESI Source Settings: Spray Voltage: 3.5 kV. Sheath Gas Flow Rate: 35 psi. Aux Gas Flow Rate: 10 L/min. Ion Transfer Tube Temperature: 320°C. S-Lens RF Level: 60. Aux Gas Heater Temperature: 350°C. Polarity: Positive and Negative. MS/MS Secondary Scans: Data-dependent scans were performed for MS/MS secondary analysis.

#### 2.7.4 Data preprocessing and metabolite identification

The raw data files (.raw) were imported into the CD 3.3 library search software for metabolite identification and quantification. Each metabolite was initially screened based on retention time, mass-to-charge ratio (m/z), and additional parameters. The peak areas were then corrected against the first QC sample to enhance the accuracy of identification. Peak extraction was performed by setting criteria including a mass deviation of 5 ppm, a signal intensity deviation of 30%, minimum signal intensity thresholds, and the inclusion of adduct ions. Target ions were integrated, and molecular formulas were predicted from molecular ion peaks and fragment ions. These predictions were compared with the mzCloud,^1^ mzVault, and Masslist databases after background ion subtraction using a blank sample. The raw quantitative results were normalized using the following formula: $\frac{\mathrm{Raw}\;\mathrm{Quantitati}\;ve\;\mathrm{Value}\;\mathrm{of}\;\mathrm{the}\;\mathrm{Sample}}{\frac{\sum \mathrm{Quantitati}\;ve\;\mathrm{Value}\;\mathrm{of}\;\mathrm{Sample}\;\mathrm{Metabolites}}{\sum \mathrm{Quantitati}\;ve\;\mathrm{Value}\;\mathrm{of}\;QC1\;\mathrm{Sample}\;\mathrm{Metabolites}\;}}$ This normalization yielded the relative peak areas. Compounds with a coefficient of variation (CV) greater than 30% in the relative peak areas of the QC samples were excluded. Following these steps, the metabolites were identified and their relative quantities were determined. Data processing was conducted on a Linux operating system (CentOS version 6.6) using R and Python software. Details of the specific packages and software versions are provided in the accompanying readme file.

### 2.8 Statistical analysis

Basic statistical analyses were conducted using GraphPad Prism version 9.5.1. For categorical data, we employed t-tests where appropriate, depending on the data characteristics. Statistical significance was determined when the two-sided *p*-value was less than 0.05. Exploratory and differential microbial composition analyses were performed using the QIIME2 software platform (Caporaso et al., 2010). In these analyses, we utilized both the actual counts of observed taxa and Shannon’s diversity index, which incorporates measures of abundance and evenness. Furthermore, to compare the pancreaticoduodenectomy group with the control group, we applied the Kruskal-Wallis test to estimate median differences between the two cohorts.

To assess beta diversity, we employed phylogenetic approaches, specifically utilizing unweighted and weighted UniFrac distances for extant or absolute data and abundance data, respectively. In addition to phylogenetic methods, we also incorporated a non-phylogenetic approach, the Bray-Curtis distance, which was similarly applied to extant or absolute data and abundance data. The Bray-Curtis distance quantifies the dissimilarity between two communities based on the presence and abundance of species. For significance testing, we conducted multivariate analysis of variance (ANOVA) to validate the robustness of our findings. This statistical method allowed us to assess the differences in community composition across the various groups while accounting for multiple variables.

Metabolite identification and pathway analysis were facilitated by consulting the KEGG database,^2^ the HMDB database,^3^ and the LIPID Maps database.^4^

Identified metabolites were annotated and subjected to multivariate statistical analysis using the metabolomics data processing software metaX (Wen et al., 2017). The data underwent principal component analysis (PCA) and partial least squares discriminant analysis (PLS-DA) to determine the variable importance in the projection (VIP) value for each metabolite. In the univariate analysis, the statistical significance (*P*-value) of each metabolite between the two groups was assessed using the t-test, and the fold change (FC-value) of metabolites between the groups was calculated. The criteria for screening differential metabolites were set as VIP > 1, *P*-value, and FC ≥ 2 or FC ≤ 0.5. Volcano plots were constructed using the R package ggplot2, which integrates the metabolite’s VIP value, log2 (FoldChange), and -log10 (*P*-value) to identify metabolites of interest. Correlation analysis, employing Pearson correlation coefficients, was performed using the cor () function in R, with statistical significance determined by cor.mtest () in R. *P*-values were considered statistically significant, and correlation plots were generated using the corrplot package in R. Bubble plots were created with the R package ggplot2 to visualize the correlations between different metabolites. The KEGG database was utilized to explore the functions and metabolic pathways of the identified metabolites. Pathways were considered enriched if the ratio of metabolites in the pathway exceeded the ratio in the entire metabolite set, and were deemed significantly enriched when the *P*-value associated with the metabolic pathway was less than 0.05.

## 3 Result

### 3.1 Subject characteristics

A total of 37 subjects were analyzed, comprising 17 patients who underwent biliary-intestinal anastomosis (Group T) and 20 sex- and age-matched subjects who did not undergo the procedure (Group N). Group T patients were further categorized into a complication group (*n* = 6) and a non-complication group (*n* = 11). The control group (Group N) consisted of 20 subjects with a male-to-female ratio of 7:13, a mean age of 63.8 years (range, 51–76 years), and a mean body mass index (BMI) of 22.7 (range, 18.3–28.2). In contrast, the biliary-intestinal anastomosis group (Group T) included 17 patients, 6 of whom experienced postoperative complications. This group had a mean age of 66 years (range, 61–75 years), a mean BMI of 21.9 (range, 19.1–26.4). The subgroup of patients who underwent biliary-intestinal anastomosis without complications included 11 subjects with a male-to-female ratio of 5:6, a mean age of 63.1 years (range, 40–79 years), and a mean BMI of 23.7 (range, 20.2–29.1) (Table 2).

**Table 2:** Table of basic information about the control group and the biliary-intestinal anastomosis group.

	Non-operated group	Biliary-intestinal anastomosis group	
		Complications	No complications
Number of subjects	20	6	11
Male/female	7/13	6/0	5/6
Age (years), mean (range)	63.8(51–76)	66(61–75)	63.1(40–79)
Body mass index, mean (range)	22.7(18.3–28.2)	21.9(19.1–26.4)	23.7(20.2–29.1)

### 3.2 Gut microbial diversity

**Figure 1:**
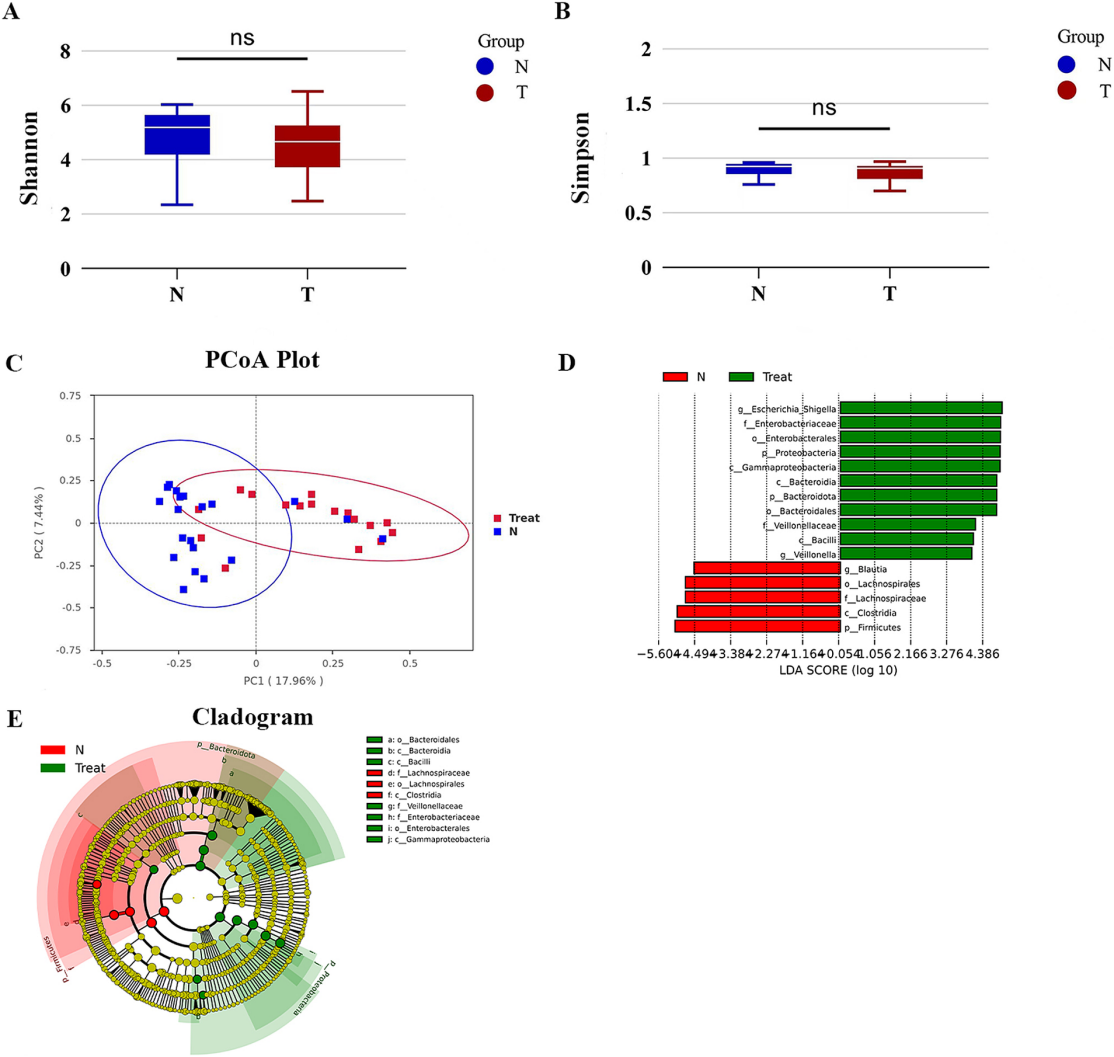
Gut microbial diversity. **(A)** Shannon index of bacterial *α*-diversity. **(B)** Simpson index of bacterial α-diversity. **(C)** *β*-diversity analysis via Principal Coordinate Analysis (PCoA) based on Bray-Curtis distances, highlighting significant compositional differences in bacterial communities between groups T and N. **(D)** LEfSe (LDA Effect Size) analysis depicting taxonomic units with the most pronounced differential abundance between groups T and N. Green bars represent taxa enriched in group T, while red bars indicate taxa enriched in group N. **(E)** Evolutionary branching diagram illustrating the statistical output of the LEfSe analysis.

We conducted a comparative analysis of fecal samples from patients who underwent biliary-intestinal anastomosis (Group T) and non-operated controls (Group N) to assess gut microbiological diversity. *α*-diversity indices, including the Shannon and Simpson indices, were calculated to evaluate the within-sample diversity of microbial communities. Although there was a tendency for these indices to decrease in Group T, the differences were not statistically significant (Figures 1A,B). For *β*-diversity analysis (Figure 1C), we employed principal coordinate analysis (pCoA) to assess the similarity between the microbial community compositions of the two groups. The Bray-Curtis distance was used to quantify the dissimilarity in microbial community composition. The plot’s first principal coordinate (PC1) and second principal coordinate (PC2) highlighted the primary distinctions between the sample sets. Each point on the graph represents an individual sample, with different colors indicating the subgroup affiliations. The pattern of sample point clustering reflected the similarity in microbial composition, while the inter-point distances visually represented the differences- greater distances indicating more pronounced compositional disparities. LEfSe (LDA Effect Size) analysis identified a total of 16 genera that exhibited potential differential abundance between group T (post-biliary-intestinal anastomosis) and group N (non-operated controls), as illustrated in Figures 1D,E.

### 3.3 Analysis of differentially abundant genera and their correlations between the biliary-intestinal anastomosis group and the non-operated control group

**Figure 2:**
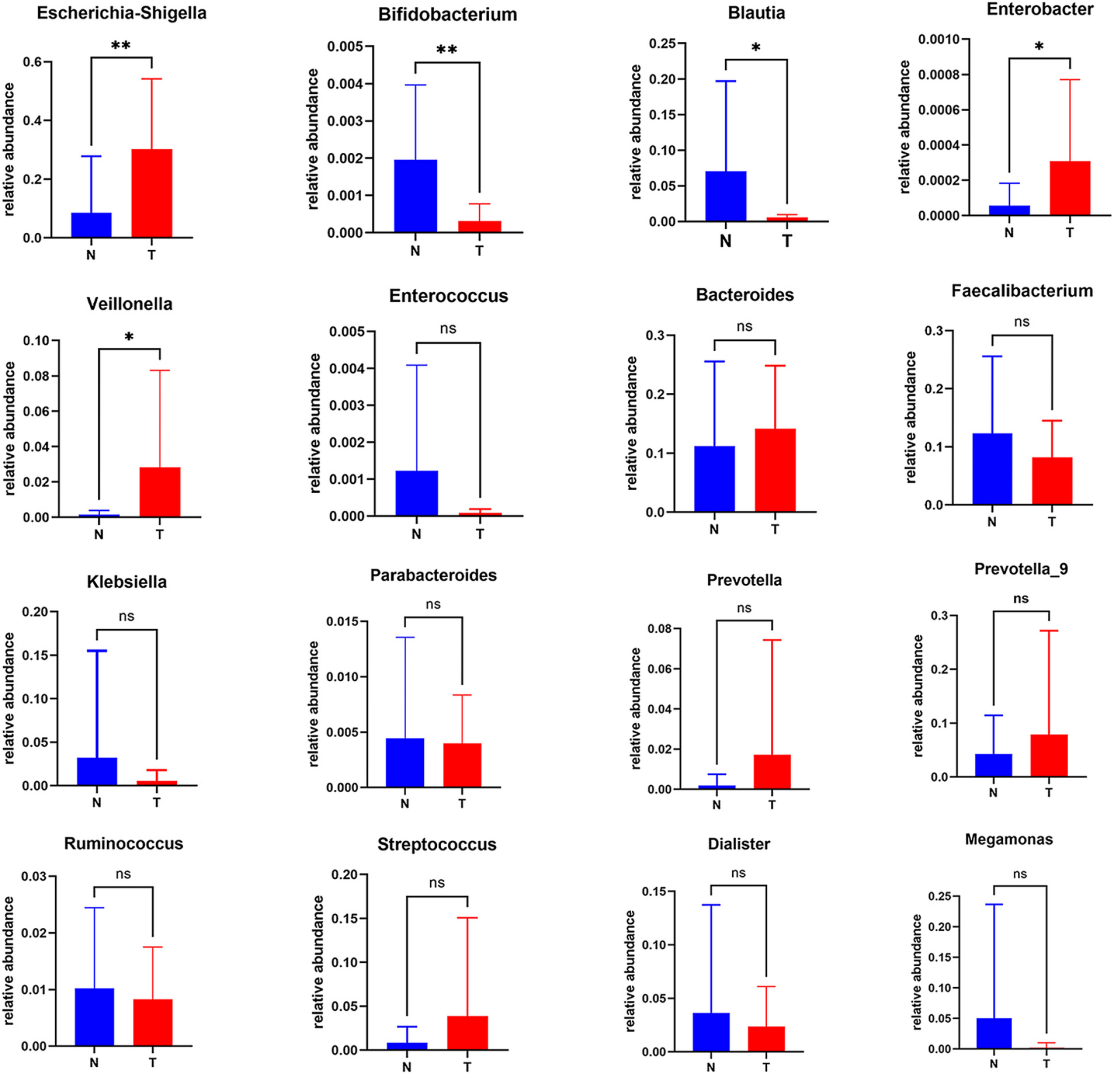
Analysis of differentially abundant genera between the biliary-intestinal anastomosis group and the unoperated control group. Out of 16 groups with notably different microbial compositions, five were identified as statistically significant following analysis. ‘ns’ signifies no statistical significance, while a single asterisk indicates *p* < 0.05 and a double asterisk indicates *p* < 0.01, indicating statistical significance.

In our comparative analysis, we focused on the top 30 genera based on their relative abundance at the genus level, uncovering statistically significant differences between the biliary-intestinal anastomosis group and the non-operated group. Upon examining a total of 16 genera with the most pronounced differences, we identified that five genera exhibited statistically significant variations (Figure 2). Specifically, Escherichia-Shigella (*P* < 0.01), Veillonella (*P* < 0.05), and Enterobacter (*P* < 0.05) demonstrated significantly higher bacterial abundance in patients post-biliary-intestinal anastomosis compared to the non-surgical group. In contrast, Blautia (*P* < 0.05) and Bifidobacterium (*P* < 0.01) showed significantly lower abundance in the surgical group compared to the non-surgical controls.

**Figure 3:**
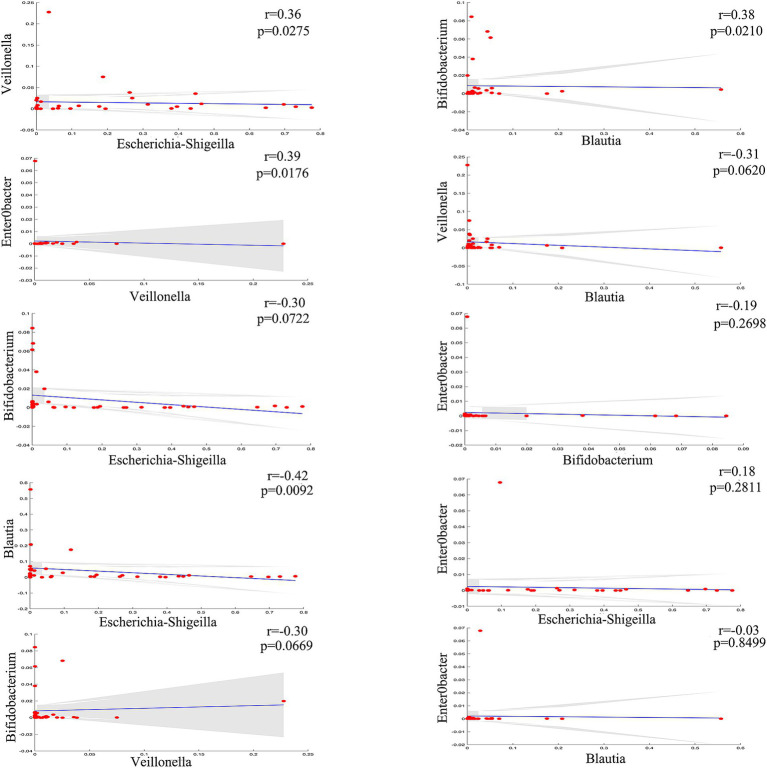
Genera correlation analysis of the biliary-intestinal anastomosis group and the non-operated control group. Correlation analysis revealed significant correlations among four groups of differentially abundant genera when compared pairwise. The correlation coefficient (r) is indicated, where positive values denote positive correlations and negative values denote negative correlations. ‘ns’ signifies no statistical significance, while a single asterisk indicates *p* < 0.05 and a double asterisk indicates *p* < 0.01, indicating statistical significance.

To further explore the correlations between the biliary-intestinal anastomosis group and the non-surgical group with respect to the significantly different flora at the genus level, we performed a Spearman correlation analysis and the degree of correlation between genera showed a significant negative correlation between Escherichia-Shigella and Blautia. Veillonella showed a significant positive correlation with both Escherichia shigella and Enterobacter. In addition, Blautia and Bifidobacterium showed a significant positive correlation with each other (Figure 3).

### 3.4 Comparative analysis of microbial flora in complication and non-complication groups

**Figure 4:**
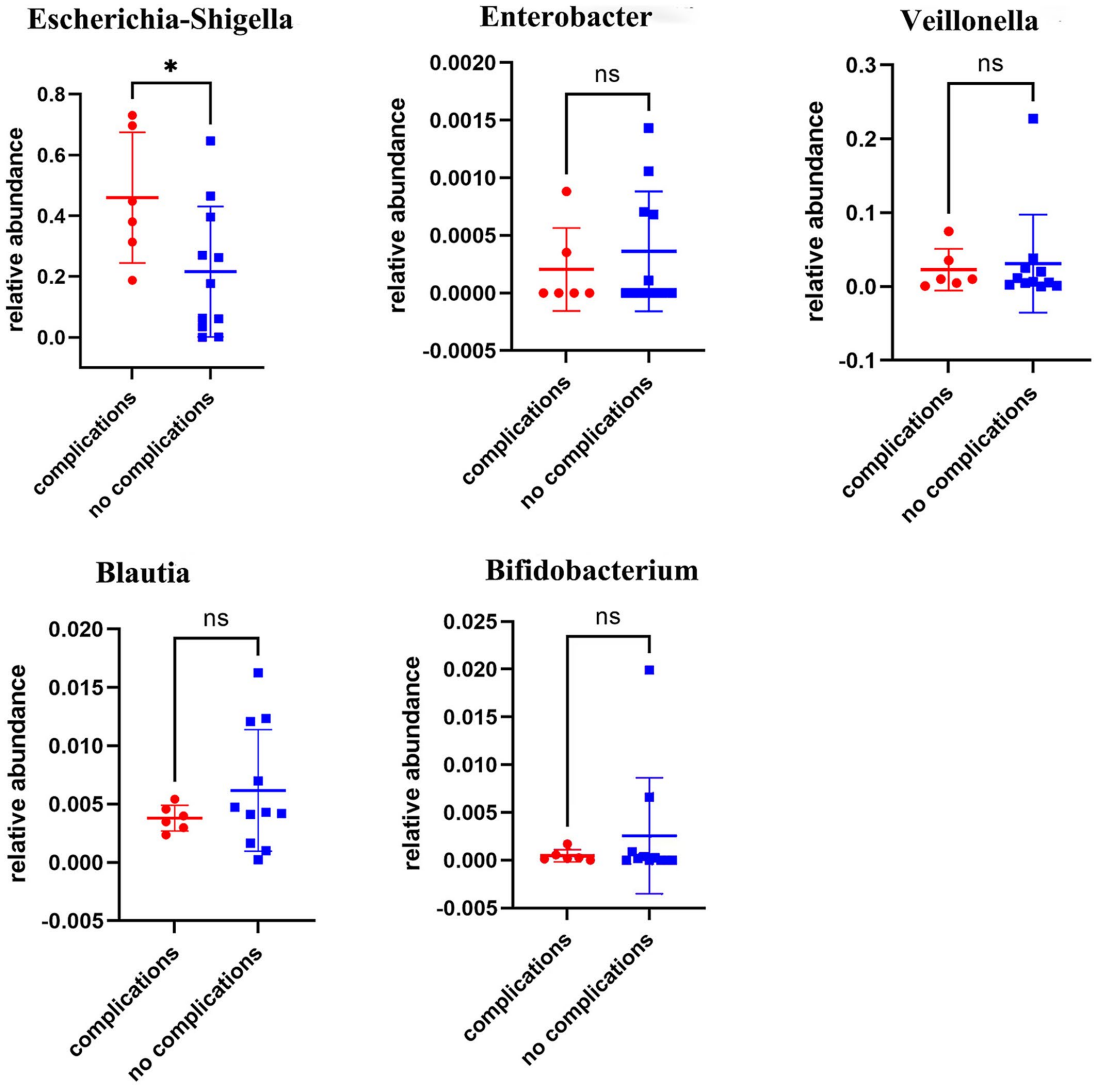
Comparative analysis of microbial flora in complication and non-complication groups. Comparative analyses revealed statistically significant differences in the abundance of Escherichia-Shigella colonies between the complication and non-complication groups, ‘ns’ signifies no statistical significance, while a single asterisk indicates *p* < 0.05.

Prior investigations have implicated alterations in the gut microbiota following biliary-intestinal anastomosis as a potential predictor of long-term complications, which may subsequently exert a detrimental influence on patient prognosis and survival. Patients who underwent biliary-intestinal anastomosis were categorized into two subgroups based on the development of postoperative complications: those with complications (complication group) and those without (non-complication group). Comparative analyses demonstrated that patients in the complication group had a significantly higher abundance of Escherichia-Shigella (*P* < 0.05) compared to the non-complication group, as depicted in Figure 4.

### 3.5 Metabolomic analysis of fecal samples: a comparative study between the biliary-intestinal anastomosis group (group T) and the non-surgical group (group N)

**Figure 5:**
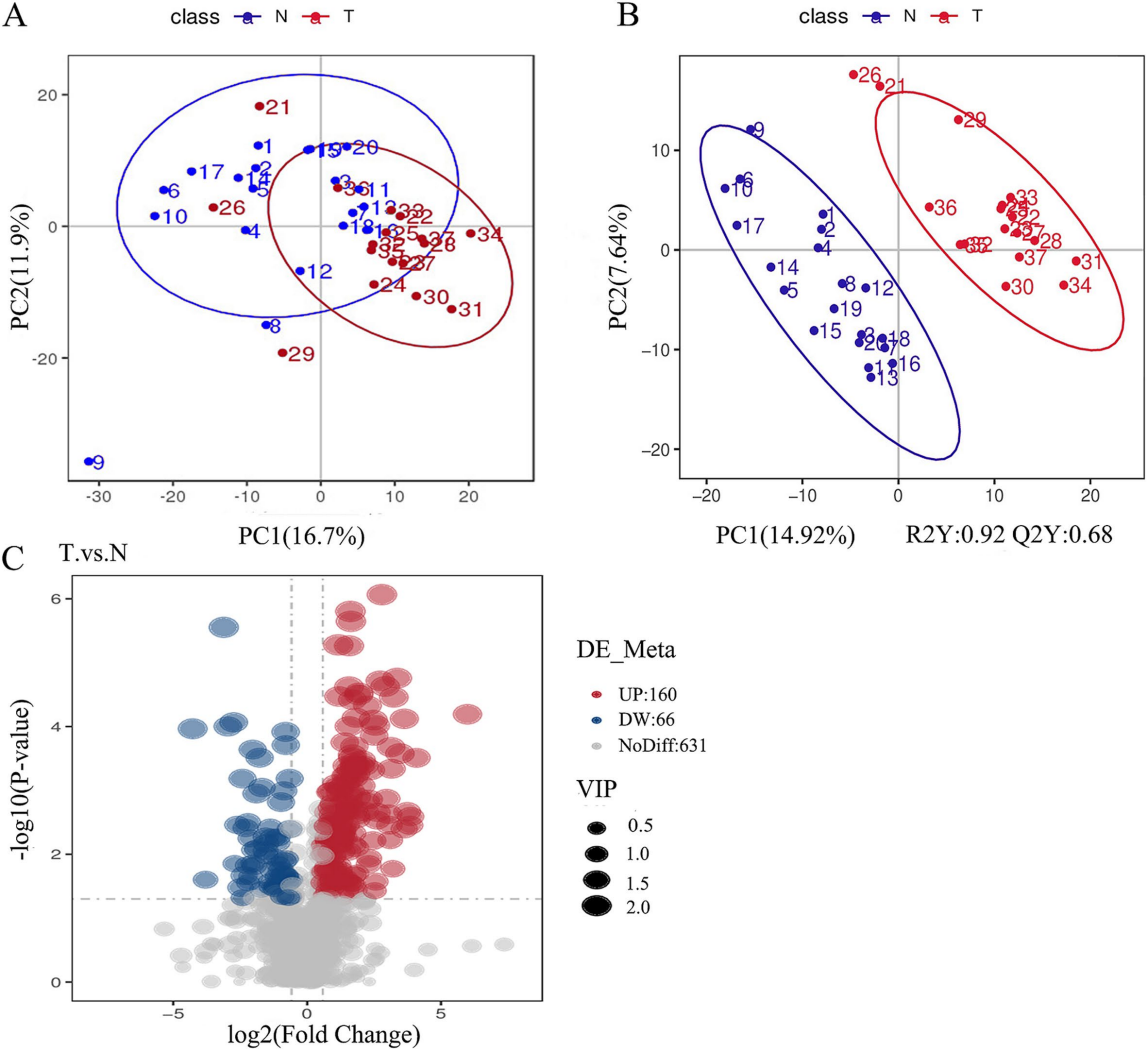
Significant differences in the metabolism of the groups were found between the biliary-intestinal anastomosis group (group T) and the non-surgical control group (group N). **(A)** A scatter plot of metabolites detected in negative ion mode following principal component analysis for dimensionality reduction. The red T-group and blue N-group show a tendency toward separation along the horizontal axis, with some overlap. The horizontal and vertical axes represent the percentage of the eigenvalues of the operational matrix relative to the total eigenvalues, indicating the degree of group differentiation. **(B)** A scatter plot derived from partial least squares discriminant analysis of the metabolites detected in negative ion mode. **(C)** A volcano plot illustrating all metabolites detected in negative ion mode, where red indicates up-regulated substances and blue indicates down-regulated substances.

Our study uncovered significant metabolic disparities between the biliary-intestinal anastomosis group (T-group) and the non-surgical group (N-group), as illustrated in Figure 5A. The distinct separation between the red N-group and the blue T-group is evident in Figure 5B, demonstrating the efficacy of the partial least squares discriminant analysis (PLS-DA) model in differentiating the two cohorts. The model’s R2Y value of 0.92 signifies a high degree of model accuracy, while the Q2Y value of 0.68 suggests a satisfactory predictive capacity. Collectively, these metrics substantiate the model’s reliability, rendering it a robust tool for the identification of differential metabolites. Figure 5C depicts the metabolites that exhibited significant differences between the biliary-intestinal anastomosis group (T-group) and the non-surgical group (N-group) based on t-test analysis, with metabolites having *P*-values less than 0.05 represented in the graph as vertical coordinates with -log10 (*P*-value) or greater. Among these significantly altered metabolites, 66 were down-regulated with a fold change (FC) value less than 0.833, while 160 were up-regulated with an FC value greater than 1.2. The size of the points in the graph corresponds to the relative contribution of each metabolite to the PLS-DA model. After stringent screening, we identified 226 substances that met the criteria of *P*-value less than 0.05, FC-value less than 0.833 or greater than 1.2, and a Variable Importance Projection (VIP) value greater than one.

**Figure 6:**
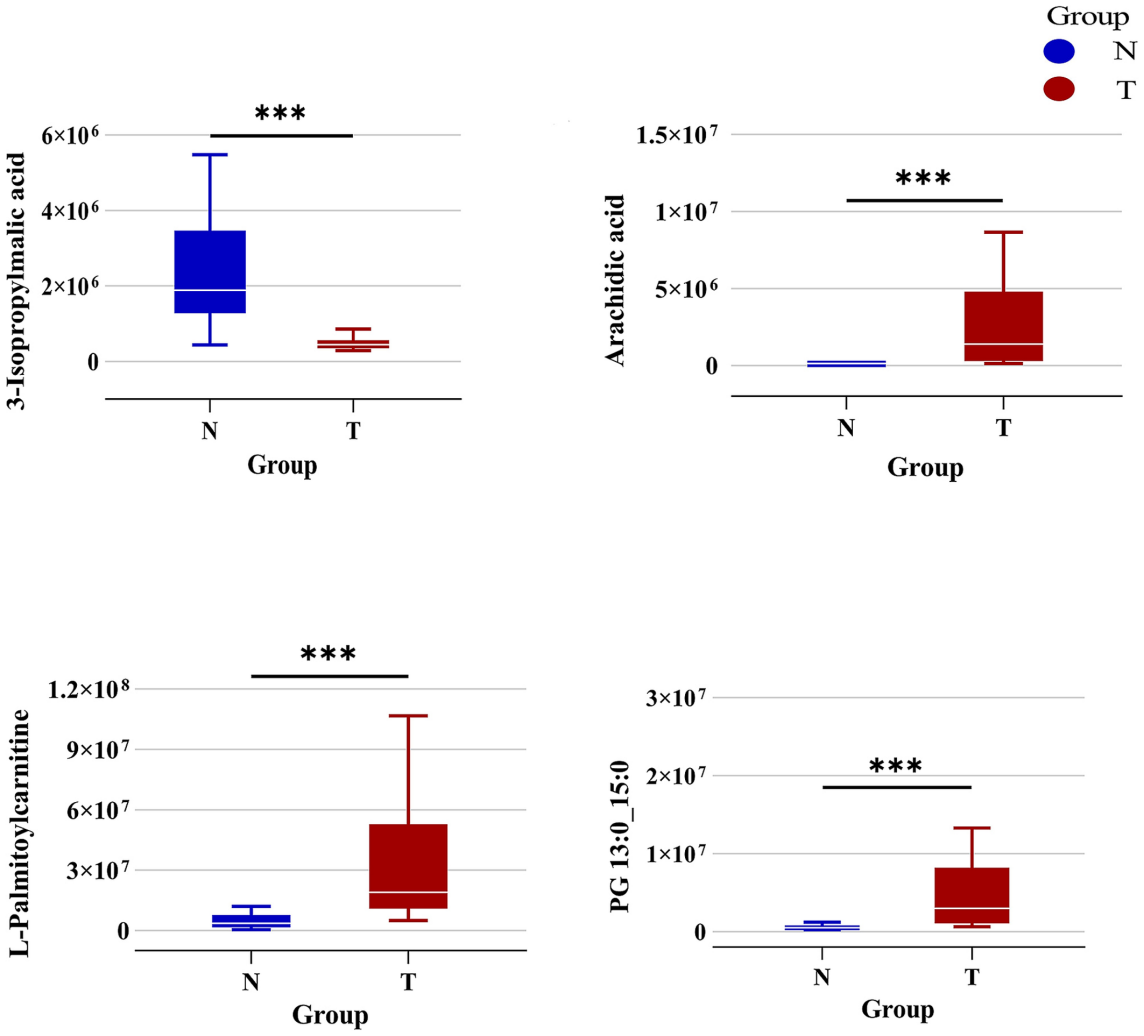
Specific differential metabolites in the biliary-intestinal anastomosis group (group T) versus the non-surgical control group (group N). Box plots representing the levels of the primary differential metabolites—L-Palmitoylcarnitine, 3-Isopropylmalic acid, Arachidic acid, and PG 13:0_15:0—between group T (red) and group N (blue). The concentrations of L-Palmitoylcarnitine, Arachidic acid, and PG 13:0_15:0 are significantly elevated in group T compared to group N. In contrast, the level of 3-Isopropylmalic acid is significantly higher in group N than in group T. These metabolites exhibit a marked difference in abundance between the two groups, indicating their potential as biomarkers of differential metabolic activity.

Additionally, we identified several key differential metabolites using selection criteria that included ROC values exceeding 0.9 and absolute logFC values surpassing 2. L-Palmitoylcarnitine, Arachidic acid, and PG 13:0_15:0 were markedly elevated in the T group compared to the N group, while 3-Isopropylmalic acid was notably reduced in the T group (Figure 6). These metabolites play a key role in the changes in the intestinal flora of patients after biliary-intestinal anastomosis.

### 3.6 KEGG pathway analysis

**Figure 7:**
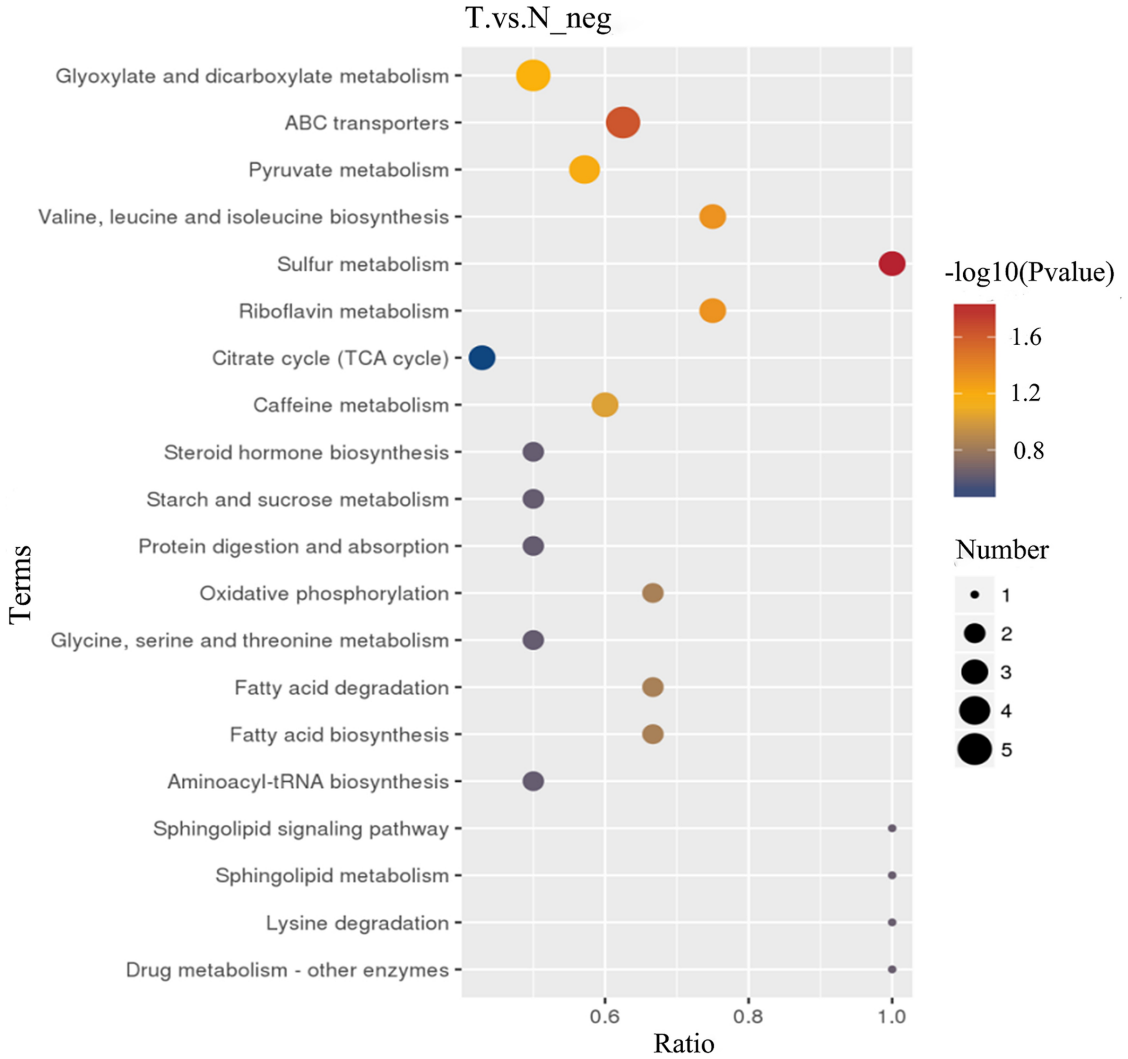
KEGG pathway analysis of metabolic differences between biliary-intestinal anastomosis group and non-surgical groups. KEGG pathway analysis revealing significant changes in nine key metabolic pathways. The colour of the dots represents the *p*-value of the pathway, and the size of the dots is positively correlated with the number of differential substances involved in this pathway. The horizontal axis, labeled ‘Ratio,’ denotes the proportion of differential metabolites involved in the pathway relative to the total number of metabolites detected within that pathway.

KEGG pathway analysis, an enrichment analysis approach, was conducted on differential metabolites detected in negative ion mode, leveraging the KEGG database for pathway mapping. Utilizing a hypergeometric distribution test, this analysis evaluated the significant overrepresentation of these differential metabolites across various metabolic pathways. As depicted in Figure 7, biliary-intestinal anastomosis induced significant alterations in multiple metabolic pathways, including starch and sucrose metabolism, steroid hormone biosynthesis, caffeine metabolism, the citric acid cycle, riboflavin metabolism, sulfur metabolism, and the biosynthesis of the branched-chain amino acids valine, leucine, and isoleucine. Moreover, the pyruvate metabolism pathway and the ABC transporter protein pathway were also significantly affected. These pathways are pivotal in the modulation of intestinal flora and its metabolic byproducts following biliary-intestinal anastomosis in patients.

## 4 Discussion

Pancreaticoduodenectomy, a procedure with extensive impact on abdominal organs, is acknowledged for its complexity and the historically high rates of postoperative complications and patient mortality. However, with advancements in surgical techniques and perioperative management, there has been a progressive reduction in the incidence of short-term postoperative complications annually. Beyond the immediate postoperative challenges such as pancreatic fistula, biliary fistula, and infections resulting from surgical trauma, the risk of long-term complications also merits significant attention. The biliary-intestinal anastomosis performed during pancreaticoduodenectomy modifies the flow of bile into the intestine, which can induce alterations in the intestinal microbiota and its metabolites. Our study revealed notable variations in the relative abundance of specific bacterial taxa—Escherichia-Shigella, Veillonella, Enterobacter, Blautia, and Bifidobacterium—and in the levels of certain metabolites, namely L-Palmitoylcarnitine, Arachidic acid, PG 13:0_15:0, and 3-Isopropylmalic acid, between patients who underwent bile-intestinal anastomosis and a non-surgical control group. These disparities may confer an elevated risk of developing long-term postoperative complications.

Escherichia-Shigella, a lipopolysaccharide-producing bacterium, has been implicated in various conditions, including cirrhosis (Tang et al., 2021), hepatocellular carcinoma, hepatitis B virus (Temraz et al., 2021), depression (Rong et al., 2019), polycystic ovary syndrome (Yu et al., 2022), and Peutz-Jeghers syndrome (Wang et al., 2022). Furthermore, alpha-fetoprotein (AFP) and serum inflammatory cytokines—interleukin-1, interleukin-6, and tumor necrosis factor-alpha—exhibit positive correlations with Escherichia-Shigella (Zhang et al., 2023). Escherichia-Shigella inversely correlates with colon length, and its dysbiosis is a hallmark of intestinal microbial imbalance (Wu et al., 2023). We propose that biliary-intestinal anastomosis, which reconstructs the digestive tract, may precipitate intestinal dysbiosis. An increase in Escherichia-Shigella can lead to the secretion of endotoxins, potentially resulting in endotoxemia and heightened inflammatory responses within the intestine. Research suggests that targeted pharmaceutical interventions can modulate the intestinal microbiota, thereby restoring the intestinal barrier and regulating inflammation. For instance, ethanol extracts have been shown to increase Bifidobacterium levels, a bacterium with anti-inflammatory properties (Li et al., 2022). Additionally, both ethanol extracts and the probiotic yeast BR14 can reduce Escherichia-Shigella, aid in the resolution of intestinal inflammation, and modulate gut barrier function (Li et al., 2022). Moreover, BR14 has been demonstrated to significantly decrease the expression of interleukin-6 and tumor necrosis factor-alpha (Mu et al., 2021).

Blautia, a dominant genus in the control group, is an anaerobic bacterium with probiotic characteristics commonly found in mammalian feces and intestines. This bacterium is known for its significant anti-inflammatory properties and plays a crucial role in maintaining intestinal health by lowering intestinal pH and elevating levels of short-chain fatty acids (SCFAs) (Liu et al., 2021). SCFAs are essential for the function of intestinal epithelial cells, serving as a primary energy source and promoting the secretion of tight junction proteins, which enhances the barrier function of the intestinal epithelium (Morrison and Preston, 2016). Furthermore, SCFAs have notable anti-inflammatory and immunomodulatory effects within the gut (van der Beek et al., 2017). Alterations in Blautia levels are indicative of changes in intestinal epithelial integrity and are widely recognized as an important biomarker for assessing intestinal damage severity (de Mooij et al., 2023). It has been established that fructooligosaccharides can effectively increase the abundance of Blautia, irrespective of dietary protein sources. Concurrently, the colonization of Blautia and Bifidobacterium (Rong et al., 2019) bifidum has been observed to diminish with age, potentially correlating with a decline in immune competence (Liu et al., 2021). Bifidobacteria are known for their beneficial immunomodulatory properties, which contribute to immune homeostasis and anti-inflammatory effects. A reduction or absence of bifidobacterial populations in humans has been strongly correlated with dysregulation of autoimmune responses and immune system imbalances (Gavzy et al., 2023).

Veillonella, an anaerobic Gram-negative coccus, is a common resident of the oral microbiome known to metabolize lactic acid and consume SCFAs, such as butyric acid (Ding et al., 2024). Notably, Veillonella enrichment has been associated with malignancy and inflammatory bowel disease, often coinciding with a decline in fecal SCFA levels (Ubachs et al., 2021). Echoing the role of Escherichia-Shigella, Mukherjee et al. proposed that elevated Veillonella levels can disrupt the intestinal microbiota balance and intensify inflammation, contributing to gastrointestinal dysfunction (Mukherjee et al., 2024). While Enterobacter species are part of the gut’s normal flora, a significant increase in Enterobacter species suggests dysbiosis post-biliary-intestinal anastomosis. We hypothesize that this surgical intervention leads to a dysregulated gut microbiome, characterized by increased abundance of Escherichia-Shigella, Veillonella, and Enterobacter, and decreased levels of Blautia and Bifidobacterium. This shift is associated with reduced SCFA production and pronounced intestinal mucosal inflammation, potentially resulting in diarrhea, impaired absorption, and a spectrum of complications. Therefore, the implementation of probiotic interventions in post-biliary anastomosis patients is crucial for maintaining intestinal microbiome equilibrium and mitigating intestinal inflammation.

Prior research has established that bile-intestinal anastomosis is frequently complicated by long-term postoperative complications, which significantly impair patient prognosis and survival, warranting close attention. Comparative analyses indicated that the complication group had a significantly higher abundance of Escherichia-Shigella. Specific probiotic interventions, including ethanol extracts and the probiotic yeast strain BR14, have demonstrated potential in mitigating Escherichia-Shigella infections, which could be instrumental in reducing the incidence of long-term complications in patients following surgery. Probiotic use following liver transplantation has been demonstrated to reduce the occurrence of bile duct-related complications (Jorgenson et al., 2018). We hypothesize that changes in the gut microbiota and its metabolites post-pancreaticoduodenectomy may significantly contribute to the development of long-term complications and that therapeutic interventions aimed at modulating these microbial imbalances and metabolites could effectively reduce the risk of such complications.

In this study, we identified significant metabolic differences in specific bacterial metabolites between the biliary-intestinal anastomosis group and the non-surgical group. L-Palmitoylcarnitine, a key intermediary in fatty acid metabolism, predominantly participates in fatty acid oxidation, and its variation may indicate disruptions in lipid metabolism (Yang J. et al., 2023). 3-Isopropylmalic acid, a metabolite in the isopropylmalic acid pathway, could be linked to the metabolic activities of specific bacterial strains. Arachidic acid, a long-chain saturated fatty acid, may reflect inflammatory reactions and alterations in cell membrane composition (Li S. et al., 2021). PG 13:0_15:0, a phospholipid integral to cell membrane structure and signaling, could influence cellular function and signaling pathways.

KEGG pathway analysis in our study revealed key metabolic pathways that significantly contribute to the gut microbiota alterations in patients following pancreaticoduodenectomy. Specifically, we observed changes in starch and sucrose metabolic pathways, which may correlate with disrupted glucose metabolism in postoperative patients. This pathway is also enriched in association with certain tumors, indicating that physiological metabolic processes are compromised during tumorigenesis (Nenkov et al., 2021). Modifications in the steroid hormone biosynthesis pathway could potentially impact hormonal balance and systemic inflammatory responses. The observed differences in the caffeine metabolism pathway might be pertinent to drug metabolism and its physiological effects. Additionally, alterations in the citric acid cycle, a hub for energy metabolism, may indicate substantial shifts in energy metabolism post-surgery. Changes in the riboflavin metabolism pathway could affect vitamin B2 utilization and its role in redox reactions. Similarly, alterations in the sulfur metabolism pathway might be linked to intracellular sulfide metabolism and antioxidant defense mechanisms. Research has demonstrated that sulfur metabolism pathways are correlated with inflammatory responses within macrophages (Takeda et al., 2023). Disturbances in the valine, leucine, and isoleucine biosynthesis pathway may reflect disruptions in amino acid metabolism among postoperative patients. Variations in the pyruvate metabolism pathway could influence glycolysis and subsequent energy metabolism. Pyruvate metabolism plays a crucial role in the activation and functionality of immune cells, and its dysregulation is associated with the development of insulin resistance and inflammatory diseases (Kim et al., 2023). Lastly, changes in the ABC transporter protein pathway might affect the transmembrane transport of substances, playing a crucial role in the functionality of the gut microbiota. These differential metabolites and the key metabolic pathways they implicate suggest that bile-intestinal anastomosis may exert impacts on lipid metabolism, inflammatory responses, and specific redox processes, potentially influencing the onset of long-term postoperative complications.

The identification of these key metabolic pathways was derived from a thorough comparative analysis of the intestinal microbiota and their metabolites between patients following pancreaticoduodenectomy and a control group that did not undergo surgery. Employing high-throughput 16S rRNA gene sequencing and KEGG pathway enrichment analyses, we observed significant alterations in these pathways. These changes reflect the intricate modulations of the intestinal microbiota and metabolic networks in postoperative patients, shedding new light on the mechanisms underlying long-term postoperative complications. Our findings not only expand our comprehension of the microbiological shifts in the postoperative intestinal environment but also pinpoint potential targets for clinical intervention. By examining the roles of these key pathways in the postoperative gut flora dynamics, we can devise more effective strategies for the prevention and management of long-term postoperative complications. This approach holds promise for enhancing patient outcomes and the quality of their survival.

## Data Availability

The original contributions presented in the study are included in the article/supplementary material, further inquiries can be directed to the corresponding author.

## Footnotes

^1^ https://www.mzcloud.org/

^2^ https://www.genome.jp/kegg/pathway.html

^3^ https://hmdb.ca/metabolites

^4^ http://www.lipidmaps.org/
